# Factors affecting the emotional wellbeing of women and men who experience miscarriage in hospital settings: a scoping review

**DOI:** 10.1186/s12884-022-04585-3

**Published:** 2022-03-31

**Authors:** Martina Galeotti, Gary Mitchell, Mark Tomlinson, Áine Aventin

**Affiliations:** 1 School of Nursing & Midwifery, Queen’s University Belfast, Belfast, Northern Ireland UK; 2 Institute for Life Course Health Research, Department of Global Health, Stellenbosch University, Cape Town, South Africa

**Keywords:** Miscarriage, Spontaneous abortion, Emotional wellbeing, Emotional distress, Emotional support, Hospital, Perinatal mental health, Scoping review

## Abstract

**Background:**

Miscarriage can be a devastating event for women and men that can lead to short- and long-term emotional distress. Studies have reported associations between miscarriage and depression, anxiety, and post-traumatic stress disorder in women. Men can also experience intense grief and sadness following their partner’s miscarriage. While numerous studies have reported hospital-related factors impacting the emotional wellbeing of parents experiencing miscarriage, there is a lack of review evidence which synthesises the findings of current research.

**Aims:**

The aim of this review was to synthesise the findings of studies of emotional distress and wellbeing among women and men experiencing miscarriage in hospital settings.

**Methods:**

A systematic search of the literature was conducted in October 2020 across three different databases (CINAHL, MEDLINE and PsycInfo) and relevant charity organisation websites, Google, and OpenGrey. A Mixed Methods appraisal tool (MMAT) and AACODS checklist were used to assess the quality of primary studies.

**Results:**

Thirty studies were included in this review representing qualitative (*N* = 21), quantitative (*N* = 7), and mixed-methods (*N* = 2) research from eleven countries. Findings indicated that women and men’s emotional wellbeing is influenced by interactions with health professionals, provision of information, and the hospital environment. Parents’ experiences in hospitals were characterised by a perceived lack of understanding among healthcare professionals of the significance of their loss and emotional support required. Parents reported that their distress was exacerbated by a lack of information, support, and feelings of isolation in the aftermath of miscarriage. Further, concerns were expressed about the hospital environment, in particular the lack of privacy.

**Conclusion:**

Women and men are dissatisfied with the emotional support received in hospital settings and describe a number of hospital-related factors as exacerbators of emotional distress.

**Implications for practice:**

This review highlights the need for hospitals to take evidence-informed action to improve emotional support services for people experiencing miscarriage within their services.

## Background

Miscarriage is the most common complication during pregnancy, affecting around 10–15% of pregnancies. Definitions of miscarriage vary across countries. In Canada, USA and Australia, for example, it is defined as a pregnancy loss occurring before the 21st week of the total 40-week gestational period, while in the United Kingdom (UK) it includes all pregnancy losses from conception up to the 24th week of gestation [1].

While it is important to acknowledge that miscarriage is not traumatic for all, there is an extensive body of literature demonstrating how women who have a miscarriage can experience short- and long-term psychological complications such as depression, anxiety and post-traumatic stress disorder [1–4]. Further, many women and men have reported experiencing intense grief as a result of their pregnancy loss [2, 3]. Experiences in hospital and, in particular, interactions with health professionals (HPs) and provision of information also have an impact on emotional wellbeing [4–6]. In fact, the quality-of-care received by women at the time of miscarriage has an impact on their mental health even years after the miscarriage has occurred [7].

Previous systematic reviews in the area have mainly focused on the psychological and emotional implications of miscarriage [8–10] and women’s satisfaction with the care provided when attending hospital due to miscarriage [11, 12]. Health professionals play a key role deliverying miscarriage care however, there is a lack of evidence demonstrating how to best support parents in hospital settings [13, 14]. Therefore, it is important to establish which factors might contribute to women and men’s emotional distress or wellbeing while in hospital, to inform provision of appropriate support and reduce the risks of developing psychological morbidities. This scoping review aimed to synthesise the evidence on hospital-related factors that contribute to parents” distress and wellbeing following miscarriage. The objectives of this review are:

- To map the available evidence and synthesise findings highlighting factors contributing to women and men’s emotional distress and wellbeing in hospital settings;
- To identify areas for further research on how best to support women and men experiencing miscarriage in hospital settings; and
- To assess the quality of the available evidence.

## Methods

This scoping review (ScR) followed guidelines from Joanna Briggs Institute (JBI), Arksey and O’Malley’s recommendations for conducting scoping reviews and the PRISMA extension for ScR to report results [15–17]. A review protocol was pre-registered on the lead author’s institutional database [18].

### Eligibility criteria for selected studies

#### Inclusion criteria

Inclusion criteria were developed using the Population, Concept and Context (PCC) framework suggested by the JBI [13].

##### Population

Studies including:

- Women and men who experienced miscarriage and attended hospital as a result.
- Health professionals’ (e.g. physicians, nurses and midwives, health care assistants, technicians, mental health professionals) with experience of working with women experiencing miscarriage in hospital settings.

##### Context

Studies referring to any hospital setting including, but not limited to, outpatients’ clinics, emergency departments and obstetrics/gynaecology wards.

##### Concept

Studies reporting hospital-related factors that contribute to the emotional distress and wellbeing of women and men experiencing miscarriage. All definitions of miscarriage were included according to the country in which the study was conducted, also recurrent miscarriage was included in the review.

##### Types of evidence sources

All types of studies, both quantitative and qualitative, published in English were included, and there were no geographical restrictions. Primary research studies, reviews, guidelines and grey literature including doctoral thesis and unpublished studies were included. Searches were restricted to the last 20 years (January 2009–October 2020).

#### Exclusion criteria

##### Population

Studies including:

- Studies of women who experience stillbirth, molar pregnancy or ectopic pregnancy and studies which do not clearly specify the type of pregnancy loss experienced.
- General practitioners, family practices and charities who have experiences of working

with women experiencing miscarriage in hospital settings.

##### Context

- Any non-hospital related factors which have influenced women and men’s emotional wellbeing.

### Search strategy

Search terms were identified in consultation with a librarian and a three-step search strategy was established. Firstly, MG used key terms (Table 1) to search CINAHL in October 2020 and, subsequently adapted this to replicate the search in MEDLINE, PsycInfo, OpenGrey and relevant international and UK based organisational websites including the ‘The Miscarriage Association’, ‘Tommy’s’, the ‘American Pregnancy Association’ and the ‘World Health Organization’ (WHO) websites. Secondly, a Google search was conducted and the results of the first five pages were screened. Thirdly, reference lists of included studies were screened for additional relevant articles.

**Table 1:** Search terms used in CINAHL

Concept/ Search terms		
Miscarriage	Psychological/emotional wellbeing	Hospital setting
Mesh headings 1. Abortion Spontaneous/ OR	Mesh headings 1. Mental Health/ OR 2. Emotions/ OR	Mesh headings 1. Health services/ OR 2. Emergency service, Hospital/ OR 3. Maternal Health Services/ Gynaecology/ OR 4. Obstetrics and Gynaecology Department, Hospital/OR 5. Obstetrics/OR
Key word 1. Miscarriage OR 2. Pregnancy loss OR 3. F?et OR 4. Early pregnancy loss OR 5. Frist trimester loss OR 6. Second trimester loss	Key word 1. Mental OR 2. Psychological OR 3. Emotional OR 4. Need* OR 5. Satisfaction* OR 6. Perspective* OR 7. Experience* OR 8. View* OR 9. Perception* OR 10. Opinion* OR	Key word 1. Healthcare OR 2. Health-care OR 3. Hospital* 4. Emergency unit* OR 5. Emergency department* OR 6. Early pregnancy unit* OR 7. Early pregnancy assessment unit*

### Study selection and data extraction

The systematic review software Covidence was used to carry out removal of duplicates, screening and data extraction. MG screened all titles and abstract and AA screened a randomly selected 10%. Further, full-text screening was completed by MG with a randomly selected 10% conducted by AA. Any disagreements throughout the process were resolved following discussion between MG and AA.

Data extraction was conducted in Covidence by MG using an adapted version of JBI Template Source of Evidence Details [19]. Studies included where charted systematically including details of the study characteristics.

### Summarising and reporting the results

Data were analysed by MG adopting the Narrative Synthesis approach [17, 18]. This method explores relationships amongst data by organising findings from included studies and describing patterns across them. MG used a diary to record and reflect on how the synthesis was conducted to promote reflexivity and transparency in the review process. Synthesis of the data took place in three phases. During data extraction, each selected study was systematically summarised using JBI Template Source of Evidence Details. This facilitated a preliminary synthesis, Phase 1, by providing details of each study in the same order and highlighting possible relationships and differences between them. During Phase 2, Nvivo Software was used to conduct thematic analysis of study findings. Specifically, initial codes were grouped in three different categories: men, women and interventions. Once this process was completed, new codes were generated by grouping together, for each category, the initial codes according to their meanings. During Phase 3, descriptive themes were created, revised and transformed into analytic themes. During this phase, the robustness of the synthesis was also assessed [19].

A Mixed Methods appraisal tool (MMAT) was used to assess the quality of primary studies. MMAT includes two screening questions applicable to all types of study designs. If studies do not pass this stage it is not feasible to proceed further with the quality appraisal. Grey literature was appraised using the AACODS checklist (Authority, accuracy, coverage, objectivity, date, significance).

## Results

**Fig. 1:**
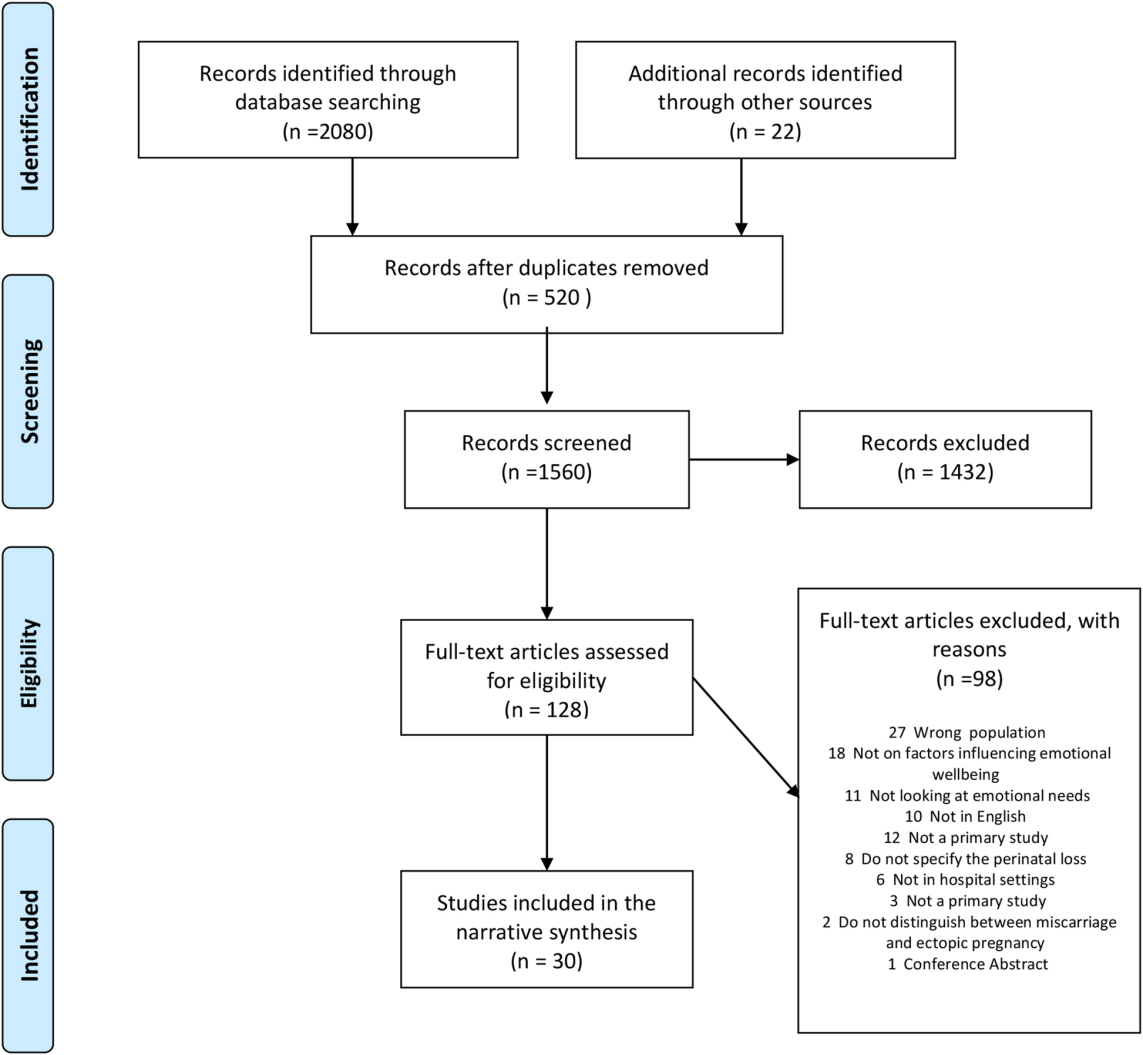
PRISMA Flow Diagram

The search yielded 2080 results and 22 articles were identified from additional sources. After removing 520 duplicates, the title and abstracts of 1560 articles were screened and a further 1432 were excluded. Finally, 128 full-text articles were screened, and thirty studies were included in this review [5, 6, 20–47]. The PRISMA flow chart summarises the above process (Fig. 1).

### Characteristics of included studies

The studies included in this review were mainly qualitative (*N* = 21), with fewer quantitative (*N* = 7), or mixed- methods (*N* = 2). Twenty- six of the studies included women (*N* = 28), a small number of studies included couples (*N* = 5), health professionals (*N* = 7) and men (*N* = 2). A total sample of 1534 people was included in the review of which 1413 were women, 38 men and 83 health professionals. Twelve studies included any pregnancy loss prior to the 24th week of gestation and did not provide information of when the miscarriage occurred. Seven studies included early pregnancy loss (up to 12 weeks), seven studies miscarriage between 5 and 16 weeks, three studies second semester miscarriage (12–19 weeks) and one study late miscarriage (20-24th weeks). One study included women who experienced solely one pregnancy loss.

The studies were conducted in 11 different countries: USA (*n* = 5), Australia (*n* = 5), United Kingdom (*n* = 5), Canada (*n* = 3), Ireland (*n* = 4), France (*n* = 2), China (*n* = 2), Brazil (*n* = 1), Denmark (*n* = 1), Poland (*n* = 1), and Thailand (*n* = 1). No studies from low or lower-middle income countries were included. A summary of the studies included can be found in Table 2.

**Table 2:** Summary of studies included in the review

Author	Country	Study design	Setting	Population	Study aim	Summary of findings
Baird et al., 2018 [48]	USA	Qualitative- interviews	Emergency Department	Women *N* = 10 Early pregnancy loss (< 13 weeks) Age range 20–41 Participant’s ethnicity Black or Afro-American 4 Hispanic 3 Other 2 White 0	To better understand why women present to Emergency Departments for early pregnancy loss and their overall experience during and after their visit.	The study identified multiple areas for improvement in quality of care, including more complete and empathetic communication, additional information, and follow up care planning. Providers in primary care and ER settings can work together to provide improved patient-centered care to women experiencing Early Pregnancy Loss.
Bellhouse at al., 2019 [5]	Australia	Qualitative- Interviews	Hospital setting -not specified	Women *N* = 15 (18–50) who experienced miscarriage 3 months to 10 years prior to interview. Age range 33–43 years old Participants’ ethnicity not disclosed in the paper	To explore women’s healthcare support experiences and how these experiences impacted women’s psychological distress following miscarriage.	Women experienced both positive and negative interactions with healthcare providers throughout their miscarriage journeys. All women interviewed expressed their increased distress following negative experiences with healthcare providers. Women commonly expressed concerns with the lack of causative information provided, a lack of follow-up from healthcare professionals, insensitive comments and terminology relating to their miscarriage, dismissive or insensitive attitudes, and a general lack of emotional support from a variety of healthcare professionals. Positive interactions with healthcare professionals included those in which women were provided with emotional support, the offer of follow-up or further testing and opportunities to express their grief through memorial services. While almost all women had some positive experiences in their interactions with healthcare professionals, most women’s stories involved significant negative experiences with healthcare providers, which caused them further distress.
Chaloumsuk, 2013 [23]	Thailand	Qualitative- Interviews with women and focus group with medical staff.	Hospital setting -not specified	Women who experienced miscarriage *N* = 11 Age range 20–30 years old Doctors *N* = 10 and nurse-midwives *N* = 11 Ethnicity: Thai 11	To gain an understanding of experiences of miscarriage and termination of pregnancy for foetal anomaly among a group of Thai women	Women need more in-depth knowledge and empathetic care from health professionals. Involving family members to support women in the labour unit can reduce the feelings of loneliness and insecurity.
Cullen et al., 2017 [49]	Republic of Ireland	Qualitative	Maternity Hospital	Women *N* = 9 and their partners *N* = 5, who experienced second semester miscarriage (15–19 weeks). Age range 30–42 years old Women’s ethnicity Irish 8 Asian 1 Men’s ethnicity Irish 4 Asian 1	To explore parents’ experiences of second trimester miscarriage and clinical care received in the hospital from the time of diagnosis through to follow-up.	Overall, the participants were very positive about how they were cared for during an extremely difficult time. However, a number of parents described negative experiences owing to insensitivity on the part of some staff, which added to their distress. Empathy and sensitivity were described by parents as ways that hospital staff recognised and helped to alleviate their suffering following a second-trimester miscarriage.
Cullen et al., 2018 [50]	Republic of Ireland	Qualitative	Maternity Hospital	Women *N* = 9 and their partners *N* = 5 who experienced second semester miscarriage (15–19 weeks). Age range 30–42 years old Women’s ethnicity Irish 8 Asian 1 Men’s ethnicity Irish 4 Asian 1	To explore parents’ experiences of second trimester miscarriage and clinical care received in the hospital from the time of diagnosis through to follow-up.	Overall, parents were satisfied with the medical treatment they received; however, some parents highlighted issues in relation to medical treatment. A number of parents commented on how busy the casualty department was and described long waits to see a doctor. Five of the women talked about difficulties in taking bloods and reported numerous attempts by staff before blood was taken successfully. The local hospital policy when a second trimester miscarriage is diagnosed is to administer mifepristone and allow the mother to go home for 48 h. Five of the women experienced this care pathway. Parents described this period of time as very difficult, but it was also acknowledged that the time allowed the parents the opportunity to begin to adjust to the loss. Being separate from pregnant women appeared to be very important to bereaved parents during outpatient appointments, casualty visits and when admitted to the hospital. Parents discussed the importance of honest and open communication with medical staff. Some parents highlighted the negative impact when communication with hospital staff was not clear.
Domingos et al., 2011 [47]	Brazil	Qualitative	Hospital setting (not specified)	Women (*N* = 13) who experienced spontaneous abortion (weeks not specified). Nurses (*N* = 7) who assisted women in the situations. Age range 18–38 Participants’ ethnicity not disclosed in the paper	To explore what is the meaning of miscarriage is for women and what they expect from HPs who care for them. To establish if there is a difference in type of care provided between public and private health care institutions. To explore how nurses feel when caring for women who experience miscarriage.	Women acknowledge that when experiencing miscarriage they require attention, support and information from the professionals. Regardless of whether they have health insurance or not, women should be treated with respect, dignity and have the right to health and citizenship guaranteed.
Edwards et al., 2018 [45]	Australia	Qualitative- interviews	Emergency Department	Women *N* = 3 and their partners **N** = 2 who presented to ED with first trimester bleeding. Nurses *N* = 6 Age of participants not disclosed in the paper Participants’ ethnicity not disclosed in the paper	To examine approaches to care provided to women who present to non-metropolitan EDS with first trimester bleeding.	The process of providing optimal care relies on the provision of nursing care that incorporates the experiences and expectation of the women and their partners as well as those nursing staff in this context.
Emond et al., 2019 [46]	Canada	Qualitative	Emergency Department	Parents who have presented to the ED with miscarriage (*N* = 14, Women = 3). ED nurses (*N* = 7) and nurse managers (*N* = 2). Average age 32.6 years old Participants’ ethnicity not disclosed in the paper	To identify the needs of parents, factors influencing their experience of care when attending ED due to miscarriage.	Parents who visit the ED during a miscarriage report multiple physical, cognitive and emotional needs. Physical health needs include a desire to undergo diagnostic testing as rapidly as possible to determine the viability of the pregnancy and to be referred for a follow-up appointment with a healthcare professional. Parents’ cognitive needs consisted of a desire to receive a detailed explanation at thetime of diagnosis, information to assist recovery, and written materials regarding the miscarriage experience and available resources and services. Finally, the emotional needs of parents include care centred on emotional health, a more private space in the ED, and, for the women experiencing miscarriage, their partner’s support.
Iwanovicz-Pakus et al., 2014 [44]	Poland	Quantitative- survey	Hospital setting (not specified)	Women who experienced miscarriage *N* = 303 Age < 20 and > 30 noted in the paper Participants’ ethnicity not disclosed in the paper	To recognise the care options for women after miscarriage in relation to support and assistance from medical staff providing care during hospitalisation.	28.71% of the women in this study admitted that they did not receive sufficient psychological support from physicians and the 22.44% definitely did not obtain such support. 41.58% of respondents reported that after miscarriage midwives showed adequate skills, and provided them with necessary informative support. Statistical analysis showed that respondents who could freely express their emotions during hospitalization evaluated physicians and midwives more positively (*p* < 0.001). Respondents who evaluated their psychological status after miscarriage as severe, expressed better evaluation of assistance and support provided by physicians (*p* < 0.001) and midwives (*p* < 0.01), compared to those who evaluated their status as moderate or light (*p* < 0.001). More than a half of the respondents needed peace and quiet during hospitalisation (58.09%), half required understanding (50.50%), nearly one-third expected seclusion (31.68%) and the same number wanted conversation (31.68%). Based on statistical analysis, the mean evaluation of the respondents’ needs during hospitalization was 15.22 ± 3.21 (5–20 scores). Respondents who evaluated their psychological status after miscarriage as severe had more intensified needs during hospitalization than the respondents who evaluated their psychological status as light or moderate (*p* < 0.0001). Respondents who at the time of pregnancy loss were married had significantly more intensified needs compare to those who were single (*p* < 0.01). The results of this study showed a significant correlation between the level of intensity of needs during hospitalisation and evaluation of physicians (*R* = 0.23; *p* = 0.00005) and midwives (*R* = 0.23; *p* = 0.02). The higher the intensity of patients’ needs, the more positive the evaluations of physicians and midwives providing them with care. Statistical analysis showed that the respondents who received complete and sufficient instructions from the medical staff concerning follow-up assistance after the loss of a baby evaluated both physicians and midwives in more positive terms, compared to those who had insufficient information or did not obtain any information at all (*p* < 0.0001).
Johnson et al., 2015 [42]	USA	RCT	Obs-Gyne Clinic and ED	40 women who experienced miscarriage between 8/40 and 20/40 weeks and attended the emergency room Age range 18–42 years old Participants’ ethnicity: Hispanic/Latino 10 African American 5 White 4 Other 1	To build an intervention to ease the potential negative consequences of grieving. The intervention was delivered in the emergency department at the time miscarriage occurred.	Findings from the study indicated that women who received the bereavement protocol reported lesser levels of overall grieving.
Klein et al., 2012 [43]	United Kingdom	Quantitative	EPAU	67 women and their partners miscarriage before 24th week of gestation IG (*N* = 33 reduced to *N* = 19 post- randomization) CG (*N* = 34 increased to *N* = 48 post-randomization) Age of participants not disclosed in the paper Participants’ ethnicity not disclosed in the paper	To establish the feasibility of undertaking a large multicentre trial using a modified PRPP design to evaluate the effectiveness of a web-based intervention in promoting the mental wellbeing of women and partners after miscarriage.	Results indicated that the IG group was significantly less anxious and depressed at the 3-month follow-up (HADS anxiety, *P* = 0.01; HADS depression, *P* = 0.02). IG group reported significantly higher levels of emotional wellbeing (SF-36 vitality, *P* = 0.018; SF-36 emotional role, *P* = 0.005; SF-36 mental health, *P* = 0.008; and SF-36 MCS score, *P* = 0.005).
Kong et al., 2013 [41]	China	RCT	Hospital setting (not specified)	180 women suffering miscarriage managed by either surgical, medical and expectant Age of participants not disclosed in the paper Participants’ ethnicity not disclosed in the paper	To investigate the clinical and psychological outcomes of surgical, medical and expectant management of first trimester miscarriage	In terms of satisfaction with the mode of treatment, there was no significant difference in the satisfaction scores between groups. Significantly more women who received either surgical or medical evacuation expressed worries of weakening or even damage to their bodies as a result of the treatment. Significantly more women with successful treatment scored higher on CSQ-8 compared with women having unsuccessful treatment. Fewer women with successful treatment expressed worries about the treatment damaging their bodies. There were no significant differences in psychological outcomes measured in terms of psychological well-being (GHQ-12), depression (BDI), anxiety (STAI) and fatigue symptoms (FS) at the time of treatment and four weeks after treatment among three treatment modalities. There was no significant correlation between randomised treatment modalities on the psychological outcome measures. Women with active intervention (both surgical and medical evacuation) had a significantly higher CIES-R score at the time of treatment when compared with women in the expectant management group. The traumatic psychological impact lessened in the subsequent follow-up at Day 28.
Kong et al., 2014 [40]	China	RCT	Hospital setting (not specified)	*N* = 280 women who were admitted to hospital with a diagnosis of miscarriage Counselling group (*N* = 140, 8 withdrawn after randomization *N* = 132). Control group *N* = 140, 4 withdrawn after randomization *N* = 136). Age of participants not disclosed in the paper Participants’ ethnicity not disclosed in the paper	To assess the effectiveness of supportive counselling after miscarriage.	A session of supportive counselling with a trained nurse counsellor, delivered immediately and at 2 weeks after diagnosis for miscarriage, did not show a statistically significant effect in reducing psychological distress of women after miscarriage. It also failed to show any additional effect. 30% reduction in the proportion of women with high GHQ-12 scores (indicative of definitive psychological distress) was evident by 3 months post miscarriage in the counselling compared with the standard care group, suggesting a potential clinical beneficial effect, albeit not a statistically significant one. Among the subset of women who had high baseline scores on the GHQ-12 and BDI questionnaires, there was a statistically significant difference was observed between counselling group and standard care groups, in terms of lower scores and reduced proportions of women scoring highly at 6 weeks in the counselling group. This suggests that a ‘selective’ counselling programme aimed at women with high baseline levels of psychological distress might be beneficial for improving emotional wellbeing in this group in the first weeks after miscarriage.
Larivière-Bastien et al., 2019 [38]	Canada	Qualitative – Interviews	Emergency Department	Women (*N* = 48) who experienced miscarriage (20 weeks or less) in the past 4 years and had consulted one of the 4 ED where the study took place. Age range 22–41 years old Participants’ ethnicity not disclosed in the paper	To identify characteristics of care management that may have contributed to the difficulties experienced by women presenting with miscarriage in the emergency department.	Analysis of the data revealed the experience of women who miscarried in the emergency department was characterized by lack of information at 3 critical junctures: announcement of the miscarriage, course of the miscarriage, and ED discharge. Respondents identified lack of information throughout the process as a recurrent factor that exacerbated the already difficult nature of this event. Although lack of information negatively influenced participants’ experiences in different ways, they shared the belief that having more information would have alleviated their difficulties. The majority of participants reported feeling unprepared emotionally and physically at the time of discharge, with long-term effects on their psychological well-being.
Linnet Olesen et al., 2015 [30]	Denmark	Qualitative- Interview	Hospital setting (not specified)	Women who experienced miscarriage and chose and completed either medical, surgical or expectant management of miscarriage. Age range 30–41 years old Participants’ ethnicity not disclosed in the paper	To gain insight into the decision-making process for the treatment of miscarriage and the circumstances that may affect it.	Unspoken emotional considerations dominated women’s reasons for choosing a specific treatment, despite pre-treatment counselling that provided detailed information about the different treatments’ efficacy and risk of side effects. Sometimes, these reasons were grounded in unrealistic beliefs about the course of the treatment. Women kept their reasons to themselves, and the HCPs did not explore them.
MacWilliams et al., 2016 [39]	Canada	Qualitative- Interview	Emergency Department	Women (*N* = 8) who had sought treatment in ED while actively miscarrying and subsequently experienced a completed miscarriage. Gestation at time of the loss 5–14 weeks. Age range 21–36 years old Participants’ ethnicity not disclosed in the paper	To explore the experience of women who attended the ED while experiencing miscarriage	Participants reported feeling isolated during discharge and after leaving the emergency department because of the lack of support and acknowledgment from HCPs, family, and friends. Having a miscarriage and receiving treatment for a miscarriage in the emergency department was a traumatic experience that had a lasting emotional impact on all the women in this study.
McLean and Flynn, 2013 [37]	Australia	Qualitative	Hospital setting (not specified)	Women (*N* = 6) who attended a hospital for a miscarriage in the first 20 weeks of pregnancy Age ranged 31–41 years old Participants’ ethnicity not disclosed in the paper	To establish a localised knowledge from a social work perspective of women’s experiences of hospital treatment after miscarriage	This study revealed that the medicalisation of miscarriage has excluded likely emotional and psychological effects from consideration. Women subsequently experience a lack of acknowledgement and compassion from the attending hospital staff, who treat them in an inconsistent, ad hoc fashion.
Meaney et al. 2017 [35]	Republic of Ireland	Qualitative	Maternity Hospital	Women (*N* = 10) and Men (*N* = 6) who experienced miscarriage (5–16 gestational weeks) Age of participants not disclosed in the paper Participants’ ethnicity not disclosed in the paper	To explore the experiences of women and men of miscarriage diagnosis and management	Analysis of the data indicated six themes in relation to the participant’s experience of miscarriage: acknowledgement of miscarriage as a valid loss, misperceptions of miscarriage, the hospital environment, management of miscarriage, support and coping, reproductive history and implications for future pregnancies.
Miller et al., 2019 [34]	USA	Mixed-methods	Emergency Department/Ambulatory settings	Women (*N* = 54) who had an ultrasound diagnosis of anembryonic gestation or embryonic or foetal demise in the first trimester (5–12 completed weeks of gestation). 1st group 25 attended the ED for miscarriage- within this group 20 completed an interview 2nd group 29 an ambulatory setting- within this group 25 completed an interview Age < 30 and > 30 noted in the paper Participants’ ethnicity Hispanic 2 Non-Hispanic 52	Sought to characterise the timeline from presentation to resolution in patients with miscarriage attending ED and ambulatory settings, as well as patient satisfaction with miscarriage management among these two groups.	The ED patient experience was qualitatively associated with greater patient confusion and less satisfaction, similar to other qualitative studies of miscarriage care in the ED where patients reported poor communication, unfriendly environment, and a lack of emotional support. Participants who sought care in the ED had longer time to miscarriage resolution and a greater number of care teams involved in the miscarriage diagnosis and management.
Miller et al. 2019 [36]	Australia	Qualitative	Hospital setting (not specified)	Men (*N* = 10) who’s partner experienced miscarriage Age range 29–49 years old Participants’ ethnicity not disclosed in the paper	To explore miscarriage from a male partner perspective, and men’s needs for additional support.	Men commonly reported a lack of emotional support from healthcare and social networks at the time of miscarriage. Men may have even less support around them at the time of miscarriage, with many stating that healthcare providers, and family and friends directed their acknowledgement and support toward their partners rather than themselves at the time of their loss. Support services and information were also largely targeted at women, leaving men feeling very isolated and alone at the time of miscarriage, and consequently they turned to online forums for support and to share their experiences of miscarriage.
Murphy and Merrell, 2009 [33]	United Kingdom	Qualitative	Gynaecological Unit	Women (*N* = 8 who had an early miscarriage Health professionals (*N* = 16) Age range 30–59 years old Participants’ ethnicity not disclosed in the paper	To explore women’s experience of having an early miscarriage in a hospital gynaecological unit.	The hospital settings emerged as highly influential in shaping the care that was given to women and influencing their experiences. Three clear phases emerged in women’s experience of miscarriage: first signs and confirmation of miscarriage, losing the baby and aftermath.
Murphy and Philpin, 2010 [6]	United Kingdom	Qualitative	Early Pregnancy Assessment Unit	Women who had an early miscarriage Health professionals (*N* = 16) Age range 30–59 years old Participants’ ethnicity not disclosed in the paper	To explore women’s experience of having an early miscarriage in a hospital gynaecological unit.	The findings presented here focused on the woman’s experience of their miscarriage with the physical symptoms of pain and blood loss being very important. There was a tension between the idea of early miscarriage as losing a baby and the actual reality of their physical experience.
Nash et al., 2018 [31]	Republic of Ireland	Qualitative	Maternity Hospital	Midwives (*N* = 8) caring for women with early pregnancy loss Age of participants not disclosed in the paper Participants’ ethnicity not disclosed in the paper	To explore the perception of midwives caring for women experiencing early pregnancy loss.	There were three main themes identified in the data analysis. These were: midwives coping with the experience of early pregnancy loss; resourcing compassionate for women; and what midwives found difficult. This study reported that exposure to early pregnancy loss can have a profound emotional effect on midwives, with a potential for this to influence the care provided to women.
Norton and Lynn, 2018 [32]	United Kingdom	Qualitative	Early Pregnancy Assessment Unit	Women (*N* = 10), who experienced miscarriage within the first 12 weeks of gestational period and had not experienced a previous miscarriage Age range 21–44 years old Participants’ ethnicity Asian 2 White 8	To explore how women experience care within an early pregnancy assessment unit and how they are helped to understand, reconcile and make sense of their loss and make informed decisions about how they care will be managed following first trimester miscarriage.	Findings from this study have some important implications for managing the care of women and their partners from the point of seeking initial help from their midwife or GP through to the provision of care in the EPAU. Individualised care is required to ensure that women and their partners do not feel ‘dehumanised’ in a system that they do not understand. The provision of individualised care, respect for women’s opinions and appropriate clinical information is imperative to those experiencing miscarriage. This is important because women respond to miscarriage differently. Furthermore, staff need to give equal consideration to women’s emotional needs as well as their physical needs to help them relieve their level of distress which in turn may help their recovery after miscarriage.
Punches et al., 2019 [29]	USA	Qualitative	Emergency Department	Women had to be between 18 and 45 years old who had experienced a pregnancy loss and were discharged home to self-care from ED department. Age range 21–34 years old Participants’ ethnicity African American 4 Caucasian 4	To describe the contemporary perspectives of women experiencing a pregnancy loss in the ED.	Women perceived that healthcare workers are withholding information and that the providers do not understand their experience. Additionally, the participants described methods of assisting with the grieving process that were beneficial, as well as some that were disconcerting.
Rowlands and Lee, 2010 [28]	Australia	Qualitative	Hospital setting (not specified)	Women (*N* = 9) who had experienced miscarriage in the previous 2 years Age range 35–42 years old Participant’s ethnicity European 6 Asian 2 Australian 2	To identify how to support Australian women after miscarriage.	Findings suggest that this lack of empathy and recognition can have a profound effect on the woman’s grieving process. Acknowledging the loss has been identified as an important part of coping with miscarriage. Two women in this study found that formal and informal ceremonies provided them with some closure and provided a meaningful way to acknowledge the loss. Acknowledging the loss was identified as an important part of coping with miscarriage. Two women in the study also found that formal and informal ceremonies provided them with some closure and provided a meaningful way to acknowledge the loss.
Schreiber et al. 2016 [27]	USA	Mixed-methods	Emergency Department	Women (55 participants in total for quantitative part 15 interviewed) who experienced miscarriage. A patient was eligible to participate if she was 1) 18 and over, 2) had an ultrasound diagnosis of an anembryonic gestation or embryonic or foetal demise in the first trimester (5–12 completed weeks of pregnancy) confirmed by 2 clinicians, neither of whom were the investigator of record, 3) willing to provide informed consent, and was 4) English speaking Age of participants not disclosed in the paper Participants’ ethnicity not disclosed in the paper	To understand patient and physician factors that might impact treatment choice and ultimate satisfaction with the goal of informing improvements in patient-centered care miscarriage care	Results suggested that women new to pregnancy rely more heavily on their clinician for guidance, and that they might benefit most substantively from care from providers with expertise in miscarriage management experiences and outcomes. Women reported the importance of having control and self-determination in concluding their miscarriage in a timely manner. Satisfaction with management is driven by the experience with the care received, rather than one specific therapeutic option over another. Satisfaction was mainly driven by efficiency of care, confidence in quality of care, sensitive providers, and effective two-way communication. Both the physician and patient-level data show alignment in considering the individual needs of the patient as well as her external demands when choosing a course of treatment.
Sejourne et al., 2010 [26]	France	Quantitative	Semi-private clinics	Women who had undergone DC or VA for the uncomplicated and anticipated loss of a pregnancy 134 women included in the study 66 assigned to one group 68 assigned to second group Age range 22–43 years old Participants’ ethnicity not disclosed in the paper	To verify if a single-session intervention based on CBT techniques including psychoeducation along with empathetic emotional support would be beneficial for women dealing with miscarriage. If CBT can be applied to preventing the psychological distress associated with a miscarriage	At 3 weeks post-miscarriage, the women in the control group had higher scores on anxiety. There were no significant differences at either 10 weeks or 6 months post-miscarriage with regards to symptom intensity. At 10 weeks, more women in the control group showed elevated scores on depression.
Smith et al. 2020 [25]	United Kingdom	Qualitative	Hospital setting (not specified)	Parents who had a miscarriage between 20 to 24th weeks of gestation. 38 parents (10 parent pairs; 18 mothers) Age of participants not disclosed in the paper Participants’ ethnicity not disclosed in the paper	To explore the healthcare experience of parents whose baby died either before, during or shortly after birth between 20 and 20th weeks of gestation in order to identify practical weys to improve healthcare provision	The key overarching theme to emerge from the interviews with parents was the importance of terminology used to refer to the death of their baby. Parents who were told they were “losing a baby” rather than “having a miscarriage” were more prepared for the realities of labour, birth experience and for making decisions around seeing and holding their baby. Appropriate terminology validated their loss, and impacted on parent’s health and wellbeing immediately following bereavement and in the long term.
Verhaeghe et al., 2020 [24]	France	Quantitative	Emergency Department	Women (*N* = 72) who had a confirmed diagnosis of first trimester pregnancy loss. Intervention group 45 consultation before the training Control group 27 consultation after the training Age range 28–36 years old Participants’ ethnicity not disclosed in the paper	To address the impact of a simulation training program for residents for the disclosure of diagnosis on the psychological experience of couples following a first trimester pregnancy loss.	Significant improvement in the couples’ personal experience and a significant decrease in the psychological morbidity associated with the disclosure following training. Attitude of the physician announcing the pregnancy loss was significantly improved, and the global perinatal grief score was significantly lower after training. Information provided was more complete and easier to understand, with fewer patients requiring an additional consultation to address uncovered issues. Overall, the study showed an objective improvement in the patients’ psychological morbidity following simulation training of the residents announcing the diagnosis, thus demonstrating a direct benefit of simulation training for patients.

### Quality of included studies

All but one [25] of the thirty studies reported receiving ethical approval. All studies used either purposive or snowball sampling, however, this is expected due to the nature of the topic under study. Seven studies had a distress protocol in place due to the sensitiveness of the topic [5, 20, 30, 31, 45–47]. Conversely, twenty-three did not mention if they had such protocol in place [6, 21–29, 32–44]. Sixteen studies declared they had no conflict of interest [5, 20–22, 28, 29, 33, 35, 37, 40–43, 45–47] and fourteen did not mention this [5, 6, 23–26, 30–32, 34, 36, 38, 39, 44]. One American and one Australian study provided compensation to participants [6, 47]. Three randomised controlled trials included had a high attrition rate [23, 37, 40]. Only one study, a doctoral thesis, was assessed using the AACODS checklist [20]. The study was assessed and considered trustworthy, accurate and meaningful.

### Synthesis of evidence

Three main themes arose from the studies: 1) interactions with health professionals; 2) The effect of time; and 3) lack of privacy in hospital environments. Within Theme 1, two sub-themes where identified 1a) Acknowledging the loss; 1b) Provision of information; 1c) Terminology. Within Theme 2, two sub-themes where identified 2a) Time waiting for diagnosis, procedures and treatment; 2b) Time to absorb the news of miscarriage. Six intervention studies were included in the review and these are discussed separately below.

### Theme 1: interactions with health professionals

#### Sub-theme 1A: acknowledging the loss

Eleven studies reported how health professionals did not acknowledge miscarriage as a loss and the extent of the emotional pain associated with it having a negative impact on parents’ emotional well-being [20, 24–26, 31, 32, 34, 36, 43, 45, 51]. These studies described how women and their partners appreciated health professionals taking time to validate and empathise with their loss [5, 25, 26, 32, 45]. Eleven studies highlighted that recognising the impact of miscarriage was considered fundamental by women and men who expressed the need to be cared for with compassion and empathy [5, 6, 20, 24–26, 31, 32, 34, 36, 43, 45].

Two studies reported that men’s experience of miscarriage was characterised by a lack of acknowledgment by health professionals who often direct their attention only towards women [34, 45]. This quote from an Australian study is representative of men’s experience “No, I was there but it wasn’t directed at me um... yeah um most of the time I was sitting there, and I was not even acknowledged” [32].

Sixteen studies described miscarriage care as lacking in emotional support where HPs neglected to explore parents’ emotions [5, 6, 20, 25–27, 29, 32–34, 36, 42, 43, 45]. Remaining unheard by HPs was a shared experience among women who reported to be unable to openly talk about their feelings. The following quote from an Australian study is representative of common experiences:

“I just think when you go in there and there is absolutely no acknowledgment of any sort of emotional thing happening, and it’s all very cut and dry” [36].

Seven other studies pointed out that women considered the emotional support received as a fundamental aspect of their hospital journey [5, 20, 25, 29, 36, 44, 45]. As expressed in this quote from a study conducted in the UK “she (the midwife) didn’t do anything medical for me, she couldn’t do anything medical for me but she did a lot more just by addressing the emotional issues related to the miscarriage” [25]. Further, two studies involving men confirmed that they expressed the same appreciation when treated with sensitivity and compassion [6, 48]. In this quote, from a study conducted in Ireland, a man explains that his miscarriage “was dealt with such good sensitivity that it made us feel a lot more comfortable … with that care, that made a bad situation that bit more bearable” [48].

#### Sub-theme 1B: provision of information

Ten studies reported lack of information throughout the course of miscarriage [25–27, 29, 34, 35, 42, 43, 45, 47]. Health professionals were perceived as being deliberately vague and unclear when providing information and this ambiguity affected women’s emotional status [23, 24, 29, 35, 48]. During miscarriage diagnosis, for instance, some studies reported that HPs did not explain clearly to women that they were miscarrying or were going to have a miscarriage [43]. For example, the following quote from a study conducted in the USA is a typical of comment made: “So it was just frustrating because it was like they were telling me the baby’s okay, then it’s not, then it’s okay, then it’s not. So it’s like a rollercoaster ride” [26]. Two studies explained that health professionals were deliberately withholding information about women’s miscarriage when communicating with women [26, 45].

Five studies reported that women left hospitals still unsure about whether they experienced a miscarriage [23, 32, 45, 46, 48]. This quote from a study based in the UK described an example of unclear communication around miscarriage “There was no baby. I only know because I looked it up. If I hadn’t looked it up I wouldn’t understand what the hell was going on; didn’t’ have a clue. That was not a good experience” [32]. Seven studies highlighted that not providing practical information on topics such as bleeding, pain, causes and prevention of miscarriage left women unprepared to deal with its aftermath [27, 35, 42, 43, 45–47]. Further, another two studies explained how women felt the need to receive extensive information about the management of miscarriage prior to making an informed decision [24, 27, 35].

#### Sub-theme 1C: terminology

Eight studies highlighted an important issue about the use of insensitive terminology and lack of lay terms used by health professionals [5, 22, 23, 25, 28, 35, 43, 47]. These studies suggested that HPs may lack compassion and sensitivity when communicating, which can increase parents’ distress. The use of what parents considered insensitive terms included “termination or abortion pills” or the word “miscarriage” when referring to late pregnancy loss and these increased women’s emotional distress [22, 28]. This quote from an Australian study reported how a man found the term “miscarriage” distressing when referring to the loss of his baby who was over 20th weeks, “because she hadn’t reached 24^th^ week, it wasn’t’ legitimate” [25].

#### Theme 2: effect of time

##### Sub-theme 2A: time waiting for diagnosis, procedures and treatment

Fifteen of the studies reported that time represented an important aspect in women’s experiences in hospital. For a variety reasons, time was relevant - time spent waiting for diagnoses, scans and procedures, or the need to be given more time to assimilate the news of miscarriage and consider treatment options [6, 20, 24–27, 29, 32, 33, 36, 42, 43, 45, 47, 48]. Having to wait in hospital while facing an unknown situation was reported to have a negative impact on women’s emotional well-being [29]. Further, waiting for long hours made women report feeling as if they were forgotten and not a priority [47, 48]. This quote from a study based in Canada is an example of what women experienced while in hospital: “There was definitely no rush, I said to myself, “Why are they making me wait until tomorrow to see the gynaecologist?” [43] Many women pointed out how waiting long hours in hospital to receive a diagnosis of miscarriage increased their anxiety [26, 48]. Further, one study noted that the longer women had to wait, the more their partners’ anger and anxiety grew and, often, this was directed towards staff members [45].

##### Sub-theme 2B: time to absorb the news of miscarriage

Two studies reported that women also required time to assimilate the news of miscarriage before moving on to deciding about their care [20, 32]. Three other studies highlighted how women appreciated a rapid resolution of miscarriage by receiving treatment on the same day they received their diagnosis [6, 36, 44].

#### Theme 3: lack of privacy in hospital environments

Five studies based at emergency departments (ED) reported that women felt distress due to a ‘chaotic’ environment. When attending the ED women had to wait in communal areas, at times with visible symptoms of miscarriage, such as bleeding, and this increased their distress [26, 33, 42, 43, 47]. Four other studies indicated that being able to access private toilet facilities was considered essential by some women who were afraid of having (or did have) their miscarriage in a shared toilet [31, 42, 43, 48]. Further, five studies reported that women expressed the need to be cared for in a private area where they could be able to be with their partners or simply away from other people [20, 29, 30, 33, 44]. This quote from a study based in Thailand described a shared feeling among women: “I cried and cried when I knew the result. A nurse took my husband and me to a small room. She said to me that my condition wasn’t urgent to treat, I and my husband could stay in this room until we felt relieved from sadness. If we needed any help, I could call her any time. This is the nursing care that I need” [20].

Four studies raised concerns on how women and their partners’ distress increased when sharing facilities with other pregnant women while in hospital [25, 29, 45, 46]. These quotes, from a woman and a man respectively, are representative of a shared experience among parents in hospital: “I was getting more and more upset ... I couldn’t really understand why the hospital didn’t have a more separated area ... It is not something a woman in any miscarriage situation should have to do”; “Going out to the toilet and there was pregnant women sitting right outside your door...you could have some kind of separate part … because that was literally the hardest thing” [48].

#### Interventions for miscarriage in hospital settings

Six intervention studies were included in this review, all of which took into consideration different aspects of miscarriage care in hospital settings [21, 23, 37–40]. Four studies targeted women [23, 37–39], one both parents [40] and one health professionals [21]. Four of these studies were behavioural interventions investigating whether psychological support offered after miscarriage might help with improving women’s emotional wellbeing [23, 37, 39]. One study based in France highlighted how communication training delivered to medical staff at the emergency department can improve parents’ mental well-being after miscarriage [21]. Another RCT highlighted that management of miscarriage per se did not impact on women’s psychological wellbeing, but the effectiveness of the treatment did [38]. A summary of the intervention findings can be found in Table 3.

**Table 3:** Summary of interventions

Author	Country	Research Design	Target Population	Intervention	Control Group	Outcomes
Verhaeghe et al. 2020 [24]	France	Uncontrolled prospective single centre before-after study	Doctors	Simulation training for doctors regarding the communication of miscarriage	None	Significant improvement in the couples’ personal experience and a significant decrease in the psychological morbidity associated with the disclosure following training. Attitude of the physician announcing the pregnancy loss was significantly improved.
Johnson et al. 2015 [42]	USA	Experimental, post-test only, control group design	Women	One-hour long bereavement sessions for women based on Guidelines for Medical Professionals Providing Care to the Family Experiencing Perinatal Loss, Neonatal Death, DIDS or other Infant Death.	Routine miscarriage care	Women who received the intervention showed less signs of grief and were 50% less likely to display despair than women in the control group. Women in both groups reported high levels of active grieving.
Kong et al. 2014 [40]	China	RCT	Women	Supportive counselling from aa single nurse counsellor in the hospital before discharge and 2 weeks later by telephone.	Routine miscarriage care	A session of supportive counselling with a trained nurse counsellor, delivered immediately and at 2 weeks after diagnosis of miscarriage, did not show a statistically significant effect in reducing the psychological distress of women after miscarriage. It also failed to show any additional effects. Among the subset of women who had high baseline scores on th Psychological wellbeing (GHQ-12) and depression (BDI) questionnaires, a statistically significant difference was observed between counselling and standard care groups in terms of lower scores and reduced proportions of women scoring highly at 6 weeks in the counselling group.
Kong et al. 2013 [41]	China	RCT	Women	Women were divided into 3 groups according to treatment chosen (medical, surgical, expectant) to measure the psychological outcomes according to treatment	N/A	Significantly more women who received either surgical or medical evacuation expressed worries of weakening or even damage to their bodies as a result of the treatment. Significantly more women with successful treatment scored higher on CSQ-8 compared with women having unsuccessful treatment. Less women with successful treatment expressed worries about the treatment damaging their bodies. There were no significant differences in psychological outcomes measured in terms of psychological well-being (GHQ-12), depression (BDI), anxiety (STAI) and fatigue symptoms (FS) at the time of treatment and four weeks after treatment among three treatment modalities. Women with active intervention (both surgical and medical evacuation) had a significantly higher score of CIES-R at the time of treatment when compared with women in the expectant management group. The traumatic psychological impact had lessened in the subsequent follow-up at Day 28.
Klein et al. 2012 [43]	United Kingdom	RCT	Parents	Women received a study pack containing participant information about “Miscarriage Matters” website and its contents (online answers from clinical experts, interactions with others as a means of support via a Forum, contact details of voluntary agencies who provide support after miscarriage)	N/A	Results indicated that the intervention group was significantly less anxious and depressed at the 3-month follow-up. Similarly, the intervention group reported significantly higher levels of emotional wellbeing.
Séjourné et al. 2010 [26]	France	RCT		All women were asked to participate in a support intervention consisting of one psychological session of CBT (cognitive behavioural therapy) on the day of the surgical intervention	No CBT session and provided with a support intervention at 3 months post-miscarriage	At 3 weeks post-miscarriage, the women in the control group had higher scores on anxiety. There were no significant differences at either 10 weeks or 6 months post-miscarriage with regards to symptom intensity. At 10 weeks, more women in the control group showed elevated scores on depression.

## Discussion

In this review we mapped the available evidence on factors contributing to women and men’s emotional distress and well-being when experiencing miscarriage in hospital settings. The evidence included strongly suggests that the care provided in hospital can negatively affect parents’ emotional wellbeing.

One of the key findings of this review was that health professionals (HPs) play a key role in shaping men and women’s experience of miscarriage and influencing their emotional wellbeing [5, 49, 52]. Being exposed to perinatal loss on a daily basis might lead HPs to view this issue as primarily medical [6, 50]. However, this review highlighted a discrepancy in perceptions of the emotional implications of miscarriage between parents and HPs, with the latter often underestimating the impact that pregnancy loss may have on women [10, 53, 54]. Parents expressed the importance of acknowledging miscarriage as a significant loss [55] and recognising both the emotional and physical implications of it [56]. Further, our review highlighted that men’s needs also need to be considered by health professionals while attending hospital with their partners as they can often feel excluded [36, 45]. These findings are in line with other systematic reviews conducted on the experience of parents’ hospital journeys [12, 57]. Four of the six interventions included in the review have shown that providing emotional support following miscarriage can contribute to the reduction of psychological morbidities in both men and women [23, 37, 39, 40]. However, all four-interventions adopted different approaches and study designs, highlighting the importance of conducting further research in this field. Also, a previous Cochrane review investigating the best psychological support following miscarriage reached “empty results” due the lack of interventions in this area [8].

Further, bereavement care guidelines emphasise the significance of communicating clearly and sensitively with women after pregnancy loss [55, 58]. Different protocols to communicate bad news stress the importance of health professionals preparing themselves before delivering difficult news by planning and providing patients with a private environment [57–60]. Further, simulation training for medical staff on how to deliver news of pregnancy loss has demonstrated a positive impact on parents, reducing psychological morbidities following miscarriage [21, 61]. However, studies in our review highlighted that both women and men are not always satisfied with how information is delivered [38, 48, 51, 62–65]. It has been reported that providing sufficient levels of information could reduce the distress associated with miscarriage [39, 62, 63]. This also arose from our findings, as the provision of information was considered essential to parents not only to promote their understanding but also their knowledge of miscarriage.

Data from this review shows the importance of using sensitive terminology when communicating to parents as many medical words such as “miscarriage” and “termination of pregnancy” can be perceived as offensive by parents. This result is in line with another review which highlighted that the use of words such as ‘fetus’ can be perceived by women as negatively impacting their emotional wellbeing [28].

Time represented an important aspect of parents’ journey. As other reviews and research studies previously highlighted, waiting while facing the unknown has a negative impact on parents’ psychological wellbeing. We further added that women identified the need for time to process the news of miscarriage before moving on to treatment options. Studies have demonstrated that miscarriage represents a traumatic event for women and their partners [46, 66]. Therefore, HPs should assess a parent’s level of understanding when delivering a diagnosis of miscarriage and ensure that enough time is provided to assimilate the news before moving on to making a shared decision about their care [67].

The results of this review suggested that it appears to be extremely distressing for parents to be in areas where privacy is lacking, for example, waiting in shared facilities with evident symptoms of miscarriage. These results mirrored another systematic reviews that highlighted the lack of private amenities accessible to women in the hospital environment [12]. Our findings highlight how important it is for women to have access to private toilets, particularly when experiencing miscarriage. Further, findings from this review indicate how parents’ distress can be increased if cared for along with other pregnant women.

### Recommendations for practice

Health professionals in hospitals and beyond should acknowledge and recognise the emotional implications of miscarriage for parents, by providing compassionate care and adequate information. Communicating empathically, without using medical terms and insensitive comments could reduce women’s anxiety while in hospital and beyond. It is also important to provide adequate time to women to process information and to give them opportunities to ask questions. The provision of training in compassionate care for HPs, in consultation with the wider community of organisations who might provide them with such, as well as considered guidelines for practice, are also urgently needed.

### Recommendations for further research

This review has revealed research which clearly indicates the need for action to improve the provision of care for those experiencing miscarriage in hospital. To guide evidence-informed guidelines and interventions for health professionals and patients, there is a need for robust research that involves the development and evaluation of evidence-based interventions. There is a particular need for interventions which can reduce the anxiety and negative emotions associated with the hospital environment. There is also a significant lack of studies involving men and same sex couples their perspectives on how they should be supported by health professionals during their partner’s miscarriage.

### Strengths and limitations

To our knowledge this is the first scoping review focusing on factors impacting on emotional wellbeing of parents who experience miscarriage and attend hospital settings. This review used a systematic process to map and assess the quality of the international literature, including primary studies and grey literature and this provided the opportunity to highlight gaps to inform practice and further research. This review also had some limitations. Only articles in English, published since 2009 were included in the review and this might have resulted in the exclusion of some relevant studies.

## Conclusions

The development of parent’s emotional distress and adjustment post-miscarriage is likely to be influenced by the emotional support provided by HPs as well as their hospital culture. None of the papers highlighted that women nor men were not emotionally distressed. This could be attributed to the fact that the main the focus of the papers was the experience of miscarriage per se and not the psychological implications of it. Further research on how to better support women in hospital settings when experiencing miscarriage is needed to inform policies and guidelines. However, this review highlighted that women and their partners need health professionals to recognise the emotional implications of miscarriage, deliver clear and effective information, and be cared for in an environment where privacy is maintained.

## Data Availability

All data generated or analysed during this study are included in this published article.
